# The Feasibility of Ultrasonographic Diaphragmatic Excursion in Healthy Dogs: Effect of Positioning, Diaphragmatic Location, and Body Weight of Dogs

**DOI:** 10.3389/fvets.2021.763556

**Published:** 2021-11-10

**Authors:** Phasamon Saisawart, Somchin Sutthigran, Kumpanart Soontornvipart, Chutimon Thanaboonnipat, Damri Darawiroj, Nan Choisunirachon

**Affiliations:** ^1^Department of Surgery, Faculty of Veterinary Science, Chulalongkorn University, Bangkok, Thailand; ^2^Department of Anatomy, Faculty of Veterinary Science, Chulalongkorn University, Bangkok, Thailand

**Keywords:** canine, diaphragm, movement, normal, ultrasound

## Abstract

Diaphragmatic excursion (DE) has been utilized for detecting respiratory related problems in humans. However, several factors should be considered such as the ultrasound technique and factors intrinsic to patients. Nevertheless, knowledge of the effect of these factors on DE in dogs is still lacking. The aim of this study was to evaluate the proper ultrasound technique by varying postures and diaphragmatic locations for DE measurement and to explore intrinsic factors such as diaphragmatic sides, sex, and body weight of dogs on DE. The prospective, analytic, cross-sectional study included 44 healthy dogs; 12 beagles and 32 dogs of other breeds. The experiment was divided into (i) an exploration of the proper ultrasound technique by varying postures (supine, standing, and recumbent in each of the right and left lateral positions), diaphragmatic locations (middle crus and proximal to the last rib), and diaphragmatic sublocations (xiphoid, mid, and proximal rib) for detection of DE and (ii) the evaluation of canine intrinsic factors affecting DE. The results show that the mid-diaphragmatic sublocation in the middle crus area in almost all positions revealed the highest percentage DE detection. However, DEs were revealed to be more accessible in the supine position. There was no significant difference in DE between the right and the left diaphragms or between the sexes of beagle dogs. However, body weight was significantly correlated with the DE among dogs of various sizes. In conclusion, the posture of the dogs and the diaphragmatic location can affect DE evaluation. Neither sex nor diaphragmatic side had an influence, but body weight was revealed as a major factor in DE in dogs.

## Introduction

Diaphragmatic excursion (DE) was first explored in roentgenography in 1969 (1) and ultrasound (US) in 1975 (2). This technique aids human medicine in the evaluation of diaphragmatic function, the major muscle function in respiration (1, 2). Initially, DE was used to detect the respiratory problems that induced dyspnea (1). In the last few years, studies have shown that DE can be utilized in several aspects of detection and clinical assistance, i.e., the detection of phrenic-nerve-injury-induced diaphragmatic paralysis (3–5), the evaluation of chronic obstructive pulmonary disease (6–8), or chronic pulmonary disease patients (9), the assessment of respiratory function in post-thoracentesis (10), or chronic kidney disease patients with hemodialysis (11), the guided technique for ventilator use and weaning in critically ill patients (12–14), and as an intervention in the diaphragmatic pacing protocol for the appropriate stimulation of diaphragmatic muscle (15–18).

Several modalities, such as thoracic radiography (19–21), US (18, 19, 22, 23), fluoroscopy (19, 24), computed tomography (19, 25), and magnetic resonance imaging (18, 19, 26, 27), have been used to detect DE. Thoracic radiography provides a high sensitivity, but it has a low specificity for detecting diaphragmatic movement (20). Fluoroscopy is a real-time imaging method that can observe diaphragmatic movement during respiratory cycles. This technique, especially in lateral recumbency, can cause a false-negative result in the evaluation of bilateral diaphragmatic paralysis due to a lack of a normal ipsilateral diaphragm for comparison (24). Computed tomography is a cross-sectional imaging modality that provides superior information to radiographs due to its tomographic nature (28). Computed tomography displays structural details of the diaphragm that are useful for an evaluation of diaphragmatic atrophy and structural abnormalities (25). However, this technique is harmful to patients because it exposes them to more ionizing radiation (29). Although magnetic resonance imaging involves no radiation, it can be used to evaluate the whole diaphragmatic motion in multiple planes (27). Magnetic resonance imaging is expensive and of limited availability. Among these modalities, US is the most common imaging modality used for the evaluation of DE in humans (23). US is widely available in veterinary practice, as it is relatively inexpensive, lacks ionizing radiation, is easy to use, shows excellent reproducibility, and provides high sensitivity and specificity (15, 18, 30–32). Furthermore, US can be used to assess diaphragmatic functions in both quantitative and qualitative evaluations (32–34). Therefore, ultrasonographic DE would be a practical technique not only for human medicine, but also for veterinary patients.

In DE evaluation, there are several factors that need to be considered. In humans, US techniques including diaphragmatic location and the angle of the US beam have been reported to influence DE (19, 33, 35, 36). Moreover, DE differs between diaphragmatic sides, and varies according to the patient's sex, age, and body weight (BW) (37–39). Although some reports revealed the utilization of DE in clinical veterinary practice, such as in the detection of diaphragmatic motion between a normal and a paralyzed diaphragm (16, 40), information of how former factors affect the healthy canine DE is still lacking. Therefore, the utilization of DE for further examinations needs to be investigated as a priority.

Given the lack of information on the proper technique of DE evaluation in dogs, including the exploration of intrinsic factors of dogs on the DE value, the objectives of this study were to compare the DE among postures in dogs and among locations of the diaphragm using healthy beagle dogs. After that, the proper postures and locations of the diaphragm selected on the basis of highest accessibility were used to evaluate the effect of diaphragmatic side, sex, and BW of dogs. We hypothesized that the posture during the DE examination in dogs and the location of the diaphragm including intrinsic factors such as the diaphragmatic side, sex, and BW would affect the DE value.

## Materials and Methods

### Animals

This study was designed as a prospective, analytic, cross-sectional study and was approved by The Institutional Animal Care and Use Committee of Chulalongkorn University, under approval number 2031027. Client-owned dogs that presented to the Diagnostic Imaging Unit, The Small Animal Hospital, Faculty of Veterinary Science, Chulalongkorn University during August 2020 and July 2021 were included in this study. All clinical information such as breed, sex including gonadal status, age, and BW was recorded. Dogs were divided into two groups: (i) healthy beagle dogs (*n* = 12) and other breed dogs (*n* = 32). The inclusion criteria for dogs in group (i) were healthy beagle dogs, of both sexes, and all gonadal statuses, with BW between 10 and 20 kg, whereas dogs in group (ii) were healthy dogs of various breeds and body sizes. All attended dogs had body condition scores of 3/5. In addition, enrolled dogs were examined to confirm their physical health condition through general appearance, mentation, hydration status, temperature, heart rate and rhythm, respiratory rate, character of mucous membrane color, capillary refill time, lung sound, and oxygen saturation in the bloodstream measured by pulse oximeter (Dash 2500, GE Medical System, USA). The criteria for a normal blood oxygen saturation level was in the ranges from 98 to 100% (41). Common hematology and basic serum biochemistry as well as thoracic and abdominal radiographs were also performed on all dogs. If any dogs had a history of diaphragmatic or cervical disease or had an experience of diaphragmatic and cervical surgery, including the dogs revealing an abnormality in the blood profile or a detectable radiographic lesion such as evidence of diaphragm abnormalities, rib fracture, mediastinal mass, cardiovascular disease, pulmonary or pleural lesions, pregnancy, intra-abdominal organomegaly, or peritoneal effusion, the dogs were excluded from the study.

### Effect of the Ultrasound Technique and Intrinsic Factors of Beagle Dogs on Diaphragmatic Excursion

**Figure 1:**
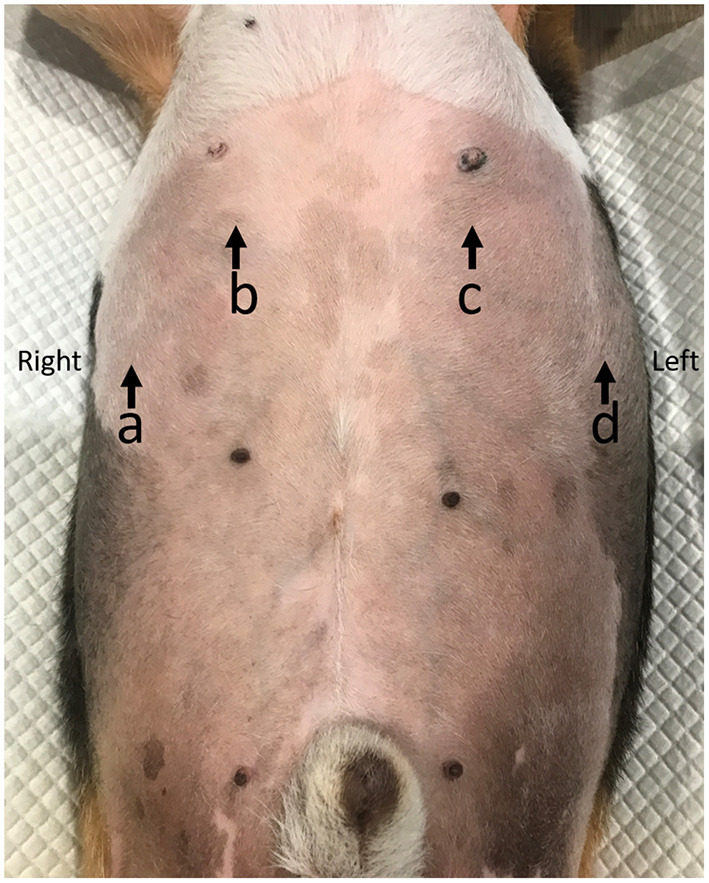
The diaphragmatic location for detecting diaphragmatic excursion in dogs. The proximal portion of the last rib of the right hemidiaphragm (PLRR; a), the mid of the right hemidiaphragmatic crus (MRHC; b), the mid of the left hemidiaphragmatic crus (MLHC; c), and the proximal portion of the last rib of the left hemidiaphragm (PLRL; d).

**Figure 2:**
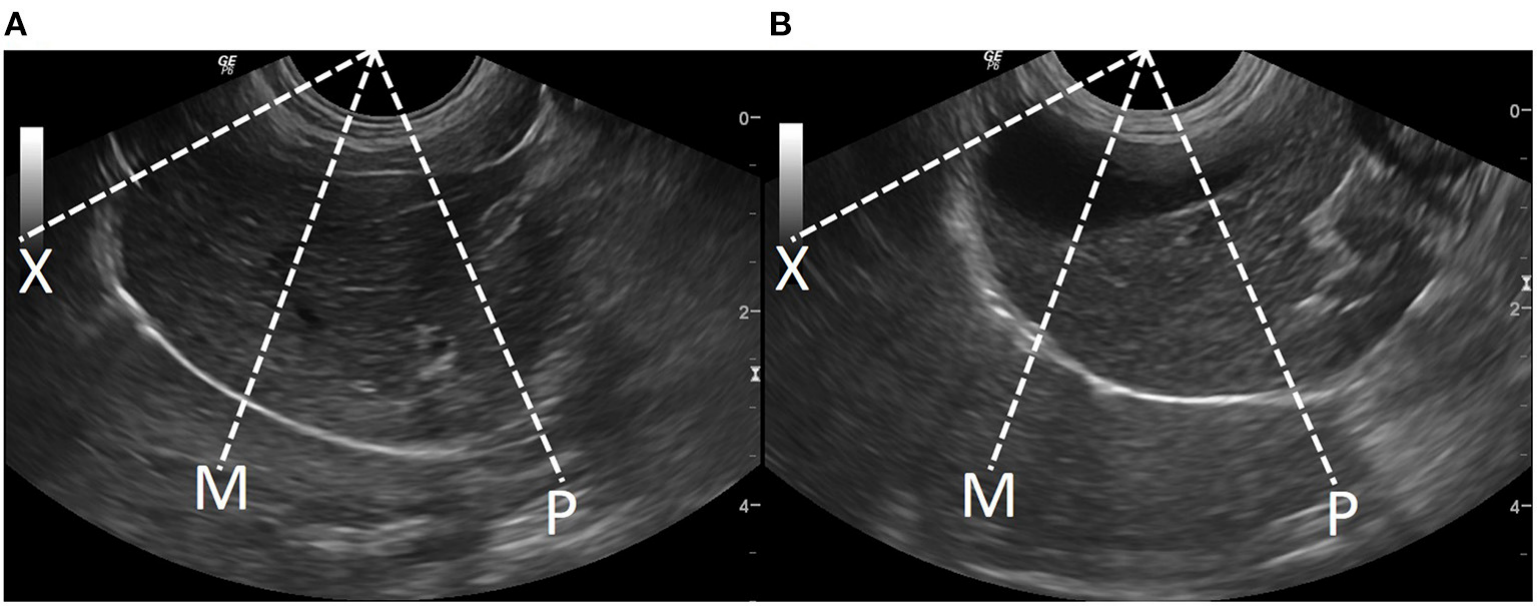
The diaphragmatic sublocation for detecting diaphragmatic excursion at left **(A)** and right **(B)** diaphragms in dogs. The distal portion of the diaphragm near the xiphoid (X), the mid of the diaphragm (M), and the proximal portion of the diaphragm near the proximal rib (P).

**Figure 3:**
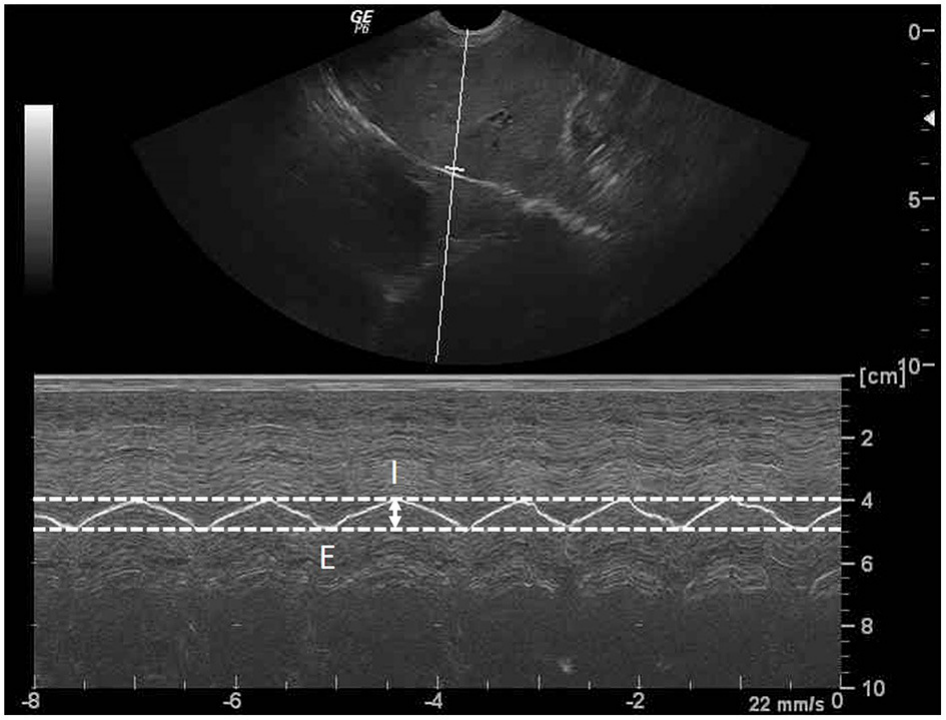
Sagittal ultrasonographic image of the diaphragm with motion (M)-mode display showed the measurement method for diaphragmatic excursion (DE; arrow). DE is the different distance of diaphragm between the peak inspiration (I) and the peak expiration (E).

All dogs included in this study were denied food and drink for 8 h. A general survey of the whole abdomen of beagle dogs using a low-frequency micro-convex transducer (7 mHz) with real time brightness mode ultrasound (Logiq P6, GE Healthcare, Korea) was performed in the supine position to assess the location of the liver and other conditions, such as distended stomach, that may affect DE. The transducer was placed in the craniodorsal direction in the sagittal plane, at the costal arch to find the hyperechoic line of each diaphragm. The liver was used as a window on the right hemidiaphragm, while the stomach or spleen was used as another window on the left hemidiaphragm and then changed from brightness to motion mode for primary evaluation of the movement of the diaphragm. To obtain a high quality ultrasound image of the diaphragm, image depth was set to ensure that the region of interest such as the diaphragmatic line was close enough for optimum visualization. The diaphragm should always be at the center about two-thirds of the field of view. Besides, a focal zone should be set only on one spot that is placed at the level of the region of interest. Diaphragmatic movement was considered normal when the diaphragm moved toward the transducer during the inspiration phase and moved outward during the expiration phase. The difference in DE between the right and the left hemidiaphragm should not be more than 50% (42). For evaluation of DE at the different locations of the diaphragm, the transducer was again placed craniodorsally in the sagittal plane at the costal arch but in different areas: (1) the proximal portion on the last rib of the right hemidiaphragm (PLRR), (2) the midpoint of the right hemidiaphragmatic crus (MRHC), (3) the midpoint of the left hemidiaphragmatic crus (MLHC), and (4) proximal portion of the last rib of the left hemidiaphragm (PLRL; Figure 1). Moreover, DE at each sublocation of the diaphragm, such as the distal portion of the diaphragm near the xiphoid with a wide US angle (xiphoid), the midpoint of the diaphragm (mid) with a perpendicular US angle, and the proximal portion of the diaphragm near the proximal rib with a narrow angle (proximal; Figure 2) were then collected on motion mode US image during spontaneous, calm breathing. US images at each location and sublocation were saved as Digital Imaging and Communication in Medicine files and viewed with Digital Imaging and Communication in Medicine viewer software (Osirix®, Geneva, Switzerland). Subsequently, DEs at each location and sublocation among different standing postures and either of the right or left lateral recumbency were additionally recorded. To measure DE, all US images were reviewed on the non-US machine monitor, and a digital caliper was applied to evaluate the different distances of the diaphragm between the peak inspiration and the peak expiration (Figure 3). All DEs at different locations and sublocations among postures were then compared.

To assess intra- and inter-observer reliabilities, US images were twice collected in beagle dogs by the same operators and between different operators, which were a well-trained, experienced master's degree student and a Thai board-certified supervisor in randomized order for each measurement.

### The Effect of Body Weight on Diaphragmatic Excursion in Dogs of Various Sizes

Following DE detection in beagle dogs, the diaphragmatic location and sublocation including the posture of the dog with the highest accessibility of DE detection was selected. Subsequently, DEs from beagle dogs were compared between sex, age, and BW, and DEs from dogs with various body sizes, including those from beagle dogs, were compared with their BWs.

### Statistical Analysis

All data were analyzed using Prism7 (GraphPad Software, CA). The normality of each data set was analyzed using the Shapiro-Wilk test. All clinical data from normal dogs were described as descriptive data expressed as means ± standard error of the mean, including median, range (minimum-maximum), and 95% confidence intervals (CI). The DEs among postures were analyzed by one-way analysis of variance (ANOVA; normal distribution) or Kruskal Wallis test (non-normal distribution), depending on the data distribution. The relationship and the associations between DE (right and left), BW, and sex were assessed using Pearson's correlation analysis. Intra-class correlation coefficient and Bland-Altman plotting were calculated for the assessment of intra- and inter-rater reliability and reported as mean ± standard deviation. All statistical analyses were significant at *P* < 0.05.

## Results

### Clinical Demographic Data

The clinical demographic information for 12 beagles and 32 dogs of other breeds is shown in Table 1. In beagle dogs, males had a slightly higher BW than females did; however, a significant difference could not be detected. Among other breed dogs, there were Pomeranian (*n* = 7), mixed breed (*n* = 4), German Shepherd (*n* = 3), Chihuahua (*n* = 3), Shih Tzu (*n* = 3), French Bulldog (*n* = 2), Poodle (*n* = 2), Welsh Corgi (*n* = 2), Yorkshire Terrier (*n* = 2), and a dog from each of the following breeds: Pug, Cairn Terrier, Pitbull, and Tibetan. The outcomes of physical examination and laboratory findings of all dogs, such as hematology and serum biochemistry, including radiographic findings and oxygen saturation levels, were within the normal reference ranges.

**Table 1:** Clinical demographic information among 12 beagles and other breed dogs.

	**Parameters**		**Beagle dogs**	**Other breed dogs**
Number			12	32
Age (years)	Mean ± SEM		3.2 ± 0.76	6.73 ± 0.67
	Median		3.15	7.50
	Range		(2.00–4.50)	(1.00–16.00)
Body weight (kg)	Mean ± SEM		14.92 ± 0.77	12.50 ± 2.30
	Median		14.00	8.50
	Range		(12.00–20.00)	(2.10–59.00)
Sex				
	Female	total	5	13
		Intact	5	8
		Neutered	0	5
	Male	total	7	19
		Intact	7	10
		Neutered	0	9

### Effect of the Ultrasound Technique on Diaphragmatic Excursion

The percent DE accessibility at different diaphragmatic locations and sublocations among postures is reported in Table 2. Among the locations and sublocations of the diaphragm, MRHC and MLHC had significantly higher DE accessibility than did PLRR and PLRL (*P* < 0.0001). Additionally, the middle of the diaphragm, especially at the MRHC and MLHC, revealed higher accessibility than the xiphoid and proximal in all postures (*P* = 0.036). Overall, the middle of the diaphragm, either at MRHC or at MLHC, revealed almost 100% DE accessibility in all postures, except in left lateral recumbency (91.67%).

**Table 2:** The accessibility of diaphragmatic excursion in beagle dogs at various locations of proximal portion of the last rib of right hemidiaphragm (PLRR), mid of the right hemidiaphragmatic crus (MRHC), mid of the left hemidiaphragmatic crus (MLHC), and proximal portion of the last rib of the left hemidiaphragm (PLRL) at various sublocations at the distal portion of the diaphragm near the xiphoid (Xiphoid), the midpoint of diaphragm (Mid), and the proximal portion of diaphragm near the proximal rib (Proximal) on supine, standing, right lateral (RL) or left lateral (LL) recumbency.

		**Accessibility of DE detection**			
**Location**	**Sublocation**	**Supine**	**Standing**	**RL**	**LL**
PLRR	Xiphoid	7/12 (58.00%)	8/12 (66.67%)	8/12 (66.67%)	12/12 (100.00%)
	Mid	7/12 (58.00%)	9/12 (75.00%)	10/12 (83.33%)	12/12 (100.00%)
	Proximal	6/12 (50.00%)	10/12 (83.33%)	5/12 (41.67%)	8/12 (66.67%)
MRHC	Xiphoid	11/12 (91.67%)	10/12 (83.33%)	12/12 (100.00%)	11/12 (91.67%)
	Mid	12/12 (100.00%)	12/12 (100.00%)	12/12 (100.00%)	11/12 (91.67%)
	Proximal	11/12 (91.67%)	10/12 (83.33%)	11/12 (91.67%)	10/12 (83.33%)
MLHC	Xiphoid	11/12 (91.67%)	12/12 (100.00%)	11/12 (91.67%)	12/12 (100.00%)
	Mid	12/12 (100.00%)	12/12 (100.00%)	12/12 (100.00%)	12/12 (100.00%)
	Proximal	9/12 (75.00%)	9/12 (75.00%)	11/12 (91.67%)	9/12 (75.00%)
PLRL	Xiphoid	8/12 (66.67%)	4/12 (33.33%)	7/12 (58.33%)	10/12 (83.33%)
	Mid	6/12 (50.00%)	3/12 (25.00%)	7/12 (58.33%)	9/12 (75.00%)
	Proximal	5/12 (41.67%)	2/12 (16.67%)	3/12 (25.00%)	7/12 (58.33.00%)

DE values detected in the different locations and sublocations were comparable in supine, left lateral, and right lateral recumbency, but not in the standing posture (*P* = 0.009; Table 3). However, the *post-hoc* difference in DEs between specific locations in the standing position could not be determined due to the variable number of accessibilities in this posture, the left hemidiaphragm revealing a slightly higher DE compared with those from the right side.

**Table 3:** The diaphragmatic excursion (cm) in beagle dogs at various locations of proximal portion of the last rib of right hemidiaphragm (PLRR), mid of the right hemidiaphragmatic crus (MRHC), mid of the left hemidiaphragmatic crus (MLHC), and proximal portion of the last rib of the left hemidiaphragm (PLRL) at various sublocations at the distal portion of the diaphragm near the xiphoid (Xiphoid), the midpoint of diaphragm (Mid), and the proximal portion of diaphragm near the proximal rib (Proximal) on the supine, standing, left lateral (LL) or right lateral (RL) recumbency.

**Location**	**Sublocation**	**Supine**	**Standing^α^**	**RL**	**LL**
PLRR	Xiphoid	0.75 ± 0.23 (0.15–2.06)	0.60 ± 0.10 (0.24–1.14)	0.84 ± 0.07 (0.50–1.06)	0.92 ± 0.12 (0.32–1.79)
	Mid	0.71 ± 0.16 (0.20–1.58)	0.57 ± 0.07 (0.17–0.90)	0.87 ± 0.09 (0.47–1.32)	0.87 ± 0.12 (0.32–1.60)
	Proximal	0.60 ± 0.12 (0.28–1.03)	0.62 ± 0.07 (0.17–0.95)	0.67 ± 0.04 (0.47–1.46)	0.63 ± 0.06 (0.38–0.95)
MRHC	Xiphoid	0.60 ± 0.08 (0.18–0.99)	0.66 ± 0.08 (0.33–1.27)	0.72 ± 0.10 (0.19–1.48)	0.63 ± 0.07 (0.26–1.01)
	Mid	0.69 ± 0.08 (0.31–1.37)	0.94 ± 0.12 (0.45–1.96)	0.86 ± 0.07 (0.45–1.18)	0.82 ± 0.07 (0.48–1.4)
	Proximal	0.73 ± 0.09 (0.34–1.43)	0.95 ± 0.12 (0.57–1.69)	0.85 ± 0.08 (0.47–1.46)	0.83 ± 0.05 (0.51–1.07)
MLHC	Xiphoid	0.68 ± 0.06 (0.49–1.07)	0.65 ± 0.12 (0.31–1.79)	0.58 ± 0.07 (0.24–0.97)	0.63 ± 0.06 (0.20–0.95)
	Mid	0.79 ± 0.10 (0.34–1.36)	1.04 ± 0.15 (0.46–2.05)	0.80 ± 0.09 (0.28–1.30)	0.79 ± 0.93 (0.42–1.14)
	Proximal	0.76 ± 0.11 (0.39–1.22)	0.97 ± 0.12 (0.51–1.57)	0.75 ± 0.09 (0.29–1.37)	0.97 ± 0.10 (0.47–1.41)
PLRL	Xiphoid	0.89 ± 0.23 (0.40–2.43)	0.77 ± 0.09 (0.56–0.99)	0.65 ± 0.05 (0.44–0.81)	0.65 ± 0.05 (0.37–1.05)
	Mid	0.82 ± 0.20 (0.31–1.73)	0.72 ± 0.14 (0.45–0.91)	0.87 ± 0.13 (0.55–1.61)	0.79 ± 0.05 (0.50–1.00)
	Proximal	0.87 ± 0.19 (0.47–1.61)	1.25 ± 0.24 (1.00–1.49)	0.97 ± 0.36 (0.53–1.70)	0.83 ± 0.11 (0.49–1.42)

α *Statistical difference between groups was made by Kruskal–Wallis test, P = 0.009*.

Due to the different accessibilities of DE among locations and sublocations, DEs at the middle of the diaphragm obtained from either MRHC or MLHC in the supine position, which were 0.69 ± 0.08 cm (95% CI = 0.31–1.37 cm and median = 0.66 cm) for the MRHC and 0.79 ± 0.10 cm (95% CI = 0.34–1.36 cm and median = 0.66 cm) for the MLHC were calculated to evaluate the ratio of DEs between the right and the left sides. The ratio of DEs between the right and the left diaphragmatic sides was 0.873.

**Figure 4:**
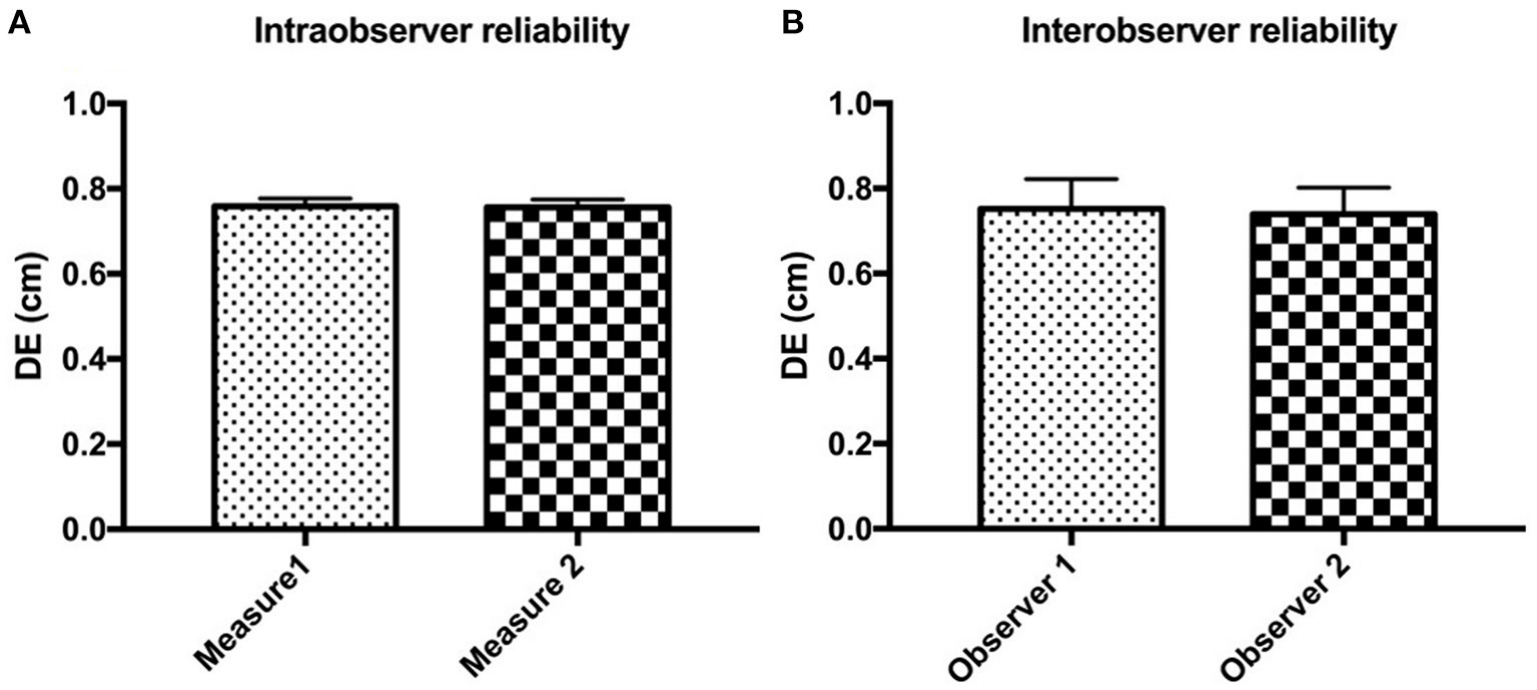
The intra- **(A)** and inter-observer **(B)** reliabilities for detecting diaphragmatic excursion (DE) in all postures, locations, and sublocations of the diaphragm in beagle dogs.

The intra- and inter-observer reliabilities for all postures, locations, and sublocations of the diaphragm are reported in Figure 4. For intra-observer reliability, the median DEs were 0.75 cm (0.19–2.05 cm) and 0.72 cm (0.20–2.05 cm) for the first and the second measurements, respectively. The concordance between two DE measurements was highly significant (*r* = 0.972, *P* < 0.001; Figure 4A). Similarly, for inter-observer reliability, the median DE values were 0.67 cm (0.50–1.40 cm) and 0.69 cm (0.48–1.37 cm) for the first and the second observers, respectively. The concordance between two measurements of DE from two observers was also highly significant (*r* = 0.919, *P* < 0.001; Figure 4B).

### The Effect of Canine Intrinsic Factors on the Diaphragmatic Excursion

**Figure 5:**
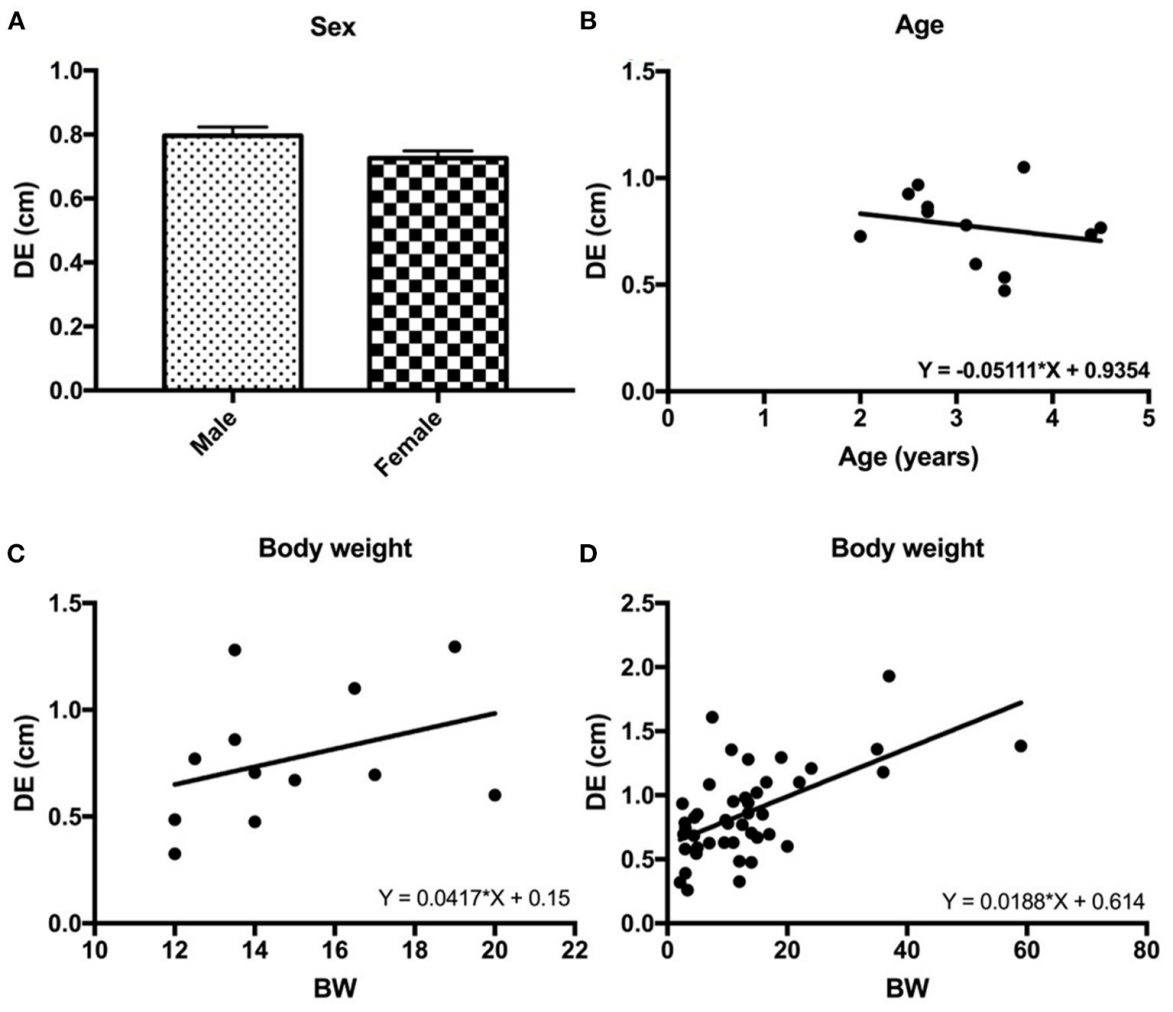
The correlations between the diaphragmatic excursion (DE) and intrinsic factors of dogs such as sex **(A)**, age **(B)**, and body weight (BW) of the beagle dogs **(C)** and BW of 32 other breed of dogs **(D)**.

In beagle dogs, although males had a slightly higher DE than females, a significant difference was not detected (*P* = 0.179; Figure 5A). Similar results for age and BW were also detected in beagle dogs in that age and BW were not significantly correlated to the DE (*R*^2^ = 0.049, *P* = 0.487 for age, Figure 5B and *R*^2^ = 0.127, *P* = 0.256 for BW, Figure 5C).

Interestingly, among different BWs and body sizes in 44 dogs, BW was significantly correlated with DE (*R*^2^ = 0.350, *P* < 0.0001; Figure 5D).

## Discussion

The diaphragm is the principal muscle of respiration. Any diseases that induce a decrease in diaphragmatic movement can cause diaphragmatic dysfunction, subsequently influencing respiration and whole-body metabolism. Recently, several reports have unveiled the advantage of DE evaluation in several abnormalities in human medicine. To utilize DE, a validated technique should be performed, and intrinsic factors of the patient that affect DE should be considered prior to diagnosis. However, this information in veterinary patients, especially in dogs, has never been explored. Therefore, a study of DE in animals by means of the proper technique and the effect of interindividual variation on the DE value would be useful information for further investigation concerning diaphragmatic movement related to respiratory abnormalities. The current study applied US to the detection of DE in dogs, and the results showed that the mid-diaphragmatic sublocation either on the MRHC or the MLHC in a supine position revealed the highest DE accessibility. On the comparable size of the dogs, DE was not affected by age and sex. However, among various sizes of dogs, BW acts as a major factor affecting the DE value. Additionally, the present study demonstrated that DE obtained by M-mode US was reproducible and repeatable. Both the intra- and inter-observer correlation coefficients were high, concordant with several previous studies in humans (15, 43, 44).

In human medicine, there are several factors that affect DE, including the pattern of respiration (15, 45), and the patient's positions during DE detection (15, 31, 43). Moreover, age, sex, and BW were also reported to affect DE values (15, 38, 45, 46). In humans, DE was reported to differ among respiratory patterns of quiet breathing, deep breathing, and voluntary sniffing (15, 31). However, since the respiratory pattern in conscious dogs could not be controlled, only the spontaneous, calm breathing pattern was selected for evaluation of the effect of other factors in this study. To explore the effect of other respiratory patterns, including respiratory disturbance from any diseases in dogs, on the DE value, further studies should be performed.

The dorsoventral dimension of the canine thoracic cavity is deeper than that of anteroposterior one in humans (47), and this factor may cause a difference in DE between dogs and humans due to both the feasibility of the detection procedure and the DE value. The present study showed that the posture of the dogs can influence the DE not only in terms of the DE value but also the examination procedure used. While a standing position was not suitable for measuring DE in dogs because it was quite difficult to restrain some restless dogs so that they remain still until the protocol was finished, lateral recumbency was revealed as a slightly easier posture for measuring DE. However, lateral recumbency was feasible for detecting DE only at the ipsilateral, upper hemidiaphragm, and it was more difficult to obtain the DE from the recumbent side. This might be due to the difficulty of placing the US transducer with an accurate point on the lower location of the cranial abdomen during lateral recumbency. In this study, DE obtained from the supine position was recommended. In the supine position, DEs from the right and the left side can be measured in one posture. Moreover, the supine position is a posture in which it is easy to restrain the animal during the examination, provides equal intra-abdominal organ distribution, and is a familiar posture for operators in several veterinary medical services. In addition, the transducer can be handled more easily to observe DE in this posture because the direction of diaphragmatic movement in the normal physiologic state is cranio-caudal movement, like a piston (32).

In addition to the examination procedure, the posture of the dog can also affect the accessibility of DE among diaphragmatic locations and sublocations. Organ and gas distribution, not only in the gastrointestinal (GI) tract but also in the pulmonary parenchyma, are the main factors affecting DE accessibility (15, 48). The US wave cannot pass through gas or bone. Therefore, an evaluation of DE may be limited in the case of gas-distended GI (49). While the accessibility of DE ranged between 91.67 and 100% in both paramedian areas (MRHC and MLHC) in all recumbents, DEs were less accessed at the proximal of the last rib areas, both at the PLRR and the PLRL, in the supine and upper ipsilateral diaphragm in the recumbent position due to interference from GI gas, such as gas in the fundus of the stomach or that in the proximal duodenum. Although in the standing posture gas is flowing dorsally away from the transducer in all diaphragmatic locations, the proximal area of the last rib was sometimes affected by GI gas. In addition, firming control of the transducer to obtain the proper DE at this diaphragmatic location on this posture is quite difficult than others. Although, the gas was flowing to the ventral abdominal wall near the transducer when the dogs were in the supine position, the small footprint that pointed into the craniodorsal direction at the MRHC and MLHC can avoid the masked gas. In addition to the diaphragmatic location, all of the xiphoid, mid, and proximal are also influential in DE detection. While pulmonary gas was often masked up the diaphragm in the ventral area near the xiphoid (15), GI gas sometimes interfered with the DE observation in the proximal area (49). The middle of the diaphragm revealed the highest accessibility in this study. Furthermore, the perpendicular direction of the craniodorsal US beam at the mid-sublocation may provide the highest accuracy of the DE detection than the others (35).

When the DE values obtained between supine and standing postures were compared, DE in the supine was higher than that in the standing position. This might have been due to the effect of gravity on the abdominal viscera (50). The displacement of abdominal organs caused wide variability of diaphragmatic movement, and the liver contributed more to this than did other abdominal organs (51). The liver falls into the cranioventral abdomen and compresses the whole diaphragm in the standing position (52). In contrast, in the supine position, the liver is relocated from the ventral to the dorsal area. This position causes the diaphragm to be less compressed by the liver than dose the standing position. Therefore, DE observed in a supine position was greater than that observed in a standing position (53).

The present DE results retrieved in lateral recumbency were comparable between the left and the right, in contrast to a previous report. DE of dogs that were observed in lateral recumbency by fluoroscopy indicated different patterns of diaphragmatic movements between right and left lateral recumbency (24). Asymmetrical diaphragmatic movement was detected in the left lateral recumbency, whereas symmetric movement of the diaphragm was detected on the right decubitus (24). The discrepancy among studies might be caused by the different techniques employed, such as different imaging modalities and different locations of DE detection on the diaphragms.

Even though DEs of the right and the left sides were not significantly different in beagle dogs, the current finding is in accordance with a previous report in dogs that the left DE seemed to be greater than that of the right (16). However, the evidence in dogs contrasted with findings from human medicine that DEs of the left side were less than those on the right side (39, 54). The liver and diaphragm are closely adjacent to each other and the movement of one is reflected in the movement of the other (53). In dogs, the greatest proportion of the liver lies to the right of the median plane, with a right-to-left proportion of liver of approximately 3:2 (55) whereas the ratio in humans ranges from 5:1 to 6:1 (56). Although the detection of DE at the right hemidiaphragm in humans is easier due to a larger window of the liver relative to the splenic window on the left side (15), DE detection on the left hemidiaphragm could be interrupted by gas in the GI, especially in the stomach. In human medicine, supine, sitting, and semi-sitting positions are commonly used (38). These postures are more comparable to supine or dorsal recumbency in dogs than other positions. An upright position such as sitting and semi-sitting in humans that gastric gas flows up to the diaphragm could cause the limited movement of the left hemidiaphragm in humans relative to that in dogs (57, 58). Although the effect is small, the gastric fundus located at the caudal area of the left hemidiaphragm can also limit the motion of the diaphragm in dogs, especially when there is gastric distension with gas and food contents. With a distended stomach, the left side of the diaphragm cannot move freely compared with the right side (54). Therefore, withholding food for the canine patient before the DE examination procedure is recommended (59).

A sex influence on DE values has been noted in several studies. In humans, it has been found that the DE of men was greater than that of women in most studies (15, 31, 39, 46) while some authors reported no statistical differences in DE between the sexes (54, 60). Moreover, significant positive correlations were found between DE of the right hemidiaphragm and BW (54, 61) and age, especially in 1 month to 2-year-old children (54). These results contrasted with our study that sex and age did not correlate to our observed DE. These might be due to the investigation in a single breed using only mature beagle dogs in the present study. Therefore, a narrow range of clinical demographic information might be a factor causing a discrepancy among studies. Interestingly, when a variety of canine body sizes were compared, BW was revealed as the major factor affecting the DE value, concordant with the results of human studies (14, 39). It has been reported that DE in normal dogs with BW ranging from 2.2 to 15.3 kg (median 5 kg) was 7.29 ± 2.24 mm (16). It seemed that differences in the DE value among studies might have been caused by differences in US procedures, such as the examination position, and detection areas by means of the diaphragmatic locations and sublocation. Therefore, these results should not be compared.

There were some limitations in this study. First, a small number of dogs were included in the study due to ethical regulations of governing the use of animals in research. Second, most of the experiments in this study were performed in beagle dogs. A large variety of dog breeds, different thoracic cage morphologies, such as a broad or narrow thorax, a dog with different body condition scores, and a comparison of normal and diseased dogs were not included. Moreover, differences in the length of the procedure period among postures were not observed and recorded. Therefore, further studies are needed to provide more information.

In conclusion, the mid-diaphragm at the middle crus or paramedian area in a supine position is the suitable area for DE detection because it provides the highest accessibility in single patient posture and is less affected by the feasibility of US examination or the gravitational distribution of the cranial abdominal organs. DE of mature dogs of a comparable size was not affected by age and sex, but BW acts as a major factor influencing the DE value. DE in dogs with various BW can be calculated by Y = 0.0188 × X + 0.614.

## Data Availability Statement

The raw data supporting the conclusions of this article will be made available by the authors, without undue reservation.

## Ethics Statement

The animal study was reviewed and approved by the Institutional Animal Care and Use Committee of Chulalongkorn University. Written informed consent was obtained from the owners for the participation of their animals in this study.

## Author Contributions

PS and NC: performed the concept/design, data analysis/interpretation, drafting article, and critical revision of article. SS: performed data analysis/interpretation, critical revision of article, and approved the article. NC: performed the data analysis as the second observer. DD, KS, and CT: performed critically revised the manuscript, and approved the article. All authors contributed to the article and approved the submitted version.

## Funding

The Scholarship from the Graduate School of Chulalongkorn University-the 90th anniversary Chulalongkorn University Fund (Ratchadaphiseksomphot Endowment Fund; Grant number GCUGR1125641046M,046).

## Conflict of Interest

The authors declare that the research was conducted in the absence of any commercial or financial relationships that could be construed as a potential conflict of interest.

## Publisher's Note

All claims expressed in this article are solely those of the authors and do not necessarily represent those of their affiliated organizations, or those of the publisher, the editors and the reviewers. Any product that may be evaluated in this article, or claim that may be made by its manufacturer, is not guaranteed or endorsed by the publisher.
